# Staphylococcus aureus food poisoning among Bulawayo City Council employees, Zimbabwe, 2014

**DOI:** 10.1186/s13104-015-1490-4

**Published:** 2015-09-28

**Authors:** Amen Gumbo, Donewell Bangure, Notion T. Gombe, More Mungati, Mufuta Tshimanga, Zanele Hwalima, Ignatious Dube

**Affiliations:** Department of Community Medicine, University of Zimbabwe, Harare, Zimbabwe; City Health Department, Bulawayo City Council, Bulawayo, Zimbabwe

**Keywords:** Food poisoning outbreak, Staphylococcus aureus, Risk factors, Council employees, Bulawayo City

## Abstract

**Background:**

Bulawayo City Council held an Integrated Result Based Management workshop among 86 employees from August 18–22, 2014 at Ikhwezi Training Centre in Bulawayo City. On August 21, 2014, a report of diarrhoea among Council employees attending the workshop was received. We investigated the outbreak to determine the risk factors associated with diarrhoea at Ikhwezi Training Centre, Bulawayo City.

**Method:**

A retrospective cohort study was conducted where 74 Council employees were interviewed on food consumed and presenting signs and symptoms. Stool specimens and hand swabs were collected for culture. Water samples were collected for bacteriological analysis. Food samples were not available. Data were analysed using Epi Info™ to generate frequencies, means, proportions, risk ratios, and attributable risk.

**Results:**

Of the 74 employees interviewed 34 (45.9 %) were males and 40 (54 %) were females. The response rate was 94 %. The common signs and symptoms included abdominal cramps (88.7 %), and watery diarrhoea (86.8 %). The overall attack rate was 71.6 %. Eating stewed chicken (RR = 2.52, 95 % CI 1.30–4.89) served at hour 13:00 during lunch on August 20, 2014 at Ikhwezi Training Centre was the only significant risk factor associated with food poisoning. Drinking purified bottled water [RR = 0.67, 95 % CI (0.57–0.79)] was found to be protective. Staphylococcus aureus was isolated from the hands and nails of food handlers.

**Conclusion:**

The outbreak was due to food poisoning and was most likely caused by the Staphylococcus aureus formed toxins. Stewed chicken served during lunch on August 20, 2014 was the possible source of infection. Contamination might have occurred during food handling and preparation. Training of food handlers in basic food hygiene and safety is recommended.

## Background

Food-borne diseases (FBDs) are defined by the World Health Organization (WHO) as “diseases of infectious or toxic nature caused by, or thought to be caused by the consumption of food or water” [1]. More than 250 FBDs have been described. Symptoms vary widely, depending on the etiological agents. Common symptoms usually include abdominal pains, diarrhoea, vomiting, mild fever headache and vomiting [1].

In 2005, approximately 1.8 million children died in developing countries from diarrhoeal diseases, which were caused by microbial agents originating from food and water. Changes in farm practices and the increasing preference for meat and poultry in developing countries have the potential to increase the incidence of foodborne illness. The extensive food distribution systems and the increasing prevalence of eating food prepared outside the home all contribute to the increased incidences of foodborne illness ascribed to microbiological organisms [1].

Acute food poisoning has also been reported in many parts of Africa. Most cases are usually mild and improve without any specific treatment. Some patients have severe disease and would require hospitalisation, aggressive rehydration and antibiotic treatment. Overall, foodborne diseases appear to cause more illnesses than deaths. Major causative agents for food poisoning in Africa include bacteria, parasites and viruses. Contamination from the environment and contaminants could also enter food during harvesting, storage, transportation and preparation for consumption [2].

The burden of foodborne disease in the African Region is difficult to surmise, however available data estimate mortality around 700,000 persons per year in all ages [3].

Disability adjusted life years lost to foodborne and waterborne disease is estimated to be 4.1 % globally compared to 5.1–7.1 % in the African region [4].

Bulawayo City Council (BCC) held an Integrated Result Based Management (IRBM) workshop for 86 employees from August 18–22, 2014 at Ikhwezi Training Centre in Bulawayo City. On August 21, 2014, a report of diarrhoea among council employees attending the workshop was made. Catering was arranged in such a way that they were served breakfast and lunch at Ikhwezi Training Centre from 18 to 22 August 2014. They did not stay overnight at the training centre and had supper at their respective residences. We investigated the outbreak to determine the risk factors, size of the outbreak as well as the presenting signs and symptoms at Ikhwezi Training Centre, Bulawayo City.

## Methods

### Epidemiological investigation

A retrospective cohort study was conducted among BCC employees attending the workshop at Ikhwezi Training Centre in Bulawayo City. A case was defined as a BCC employee attending the IRBM Training at Ikhwezi Training Centre from August 18–22, 2014, who had gastrointestinal illness between 3 p.m. on August 20, 2014 and 10 a.m. on August 21, 2014. A minimum sample size of 79 was calculated. Convenient sampling of respondents was done. A pretested interviewer administered questionnaire structured from the WHO guidelines for investigating food poisoning outbreaks was used [5]. The information asked for in the questionnaire included demographic information such as age and sex, clinical information such as date and time of first signs and symptoms and risk information such as a detailed food history. A different interviewer administered questionnaire was used to obtain information from food handlers.

### Laboratory investigation

Swabs were taken from the hands and nails of food handlers who were involved in the preparation of food consumed by council employees. Swabs and stool specimens were collected between 11 a.m. and 12 p.m. on August 21, 2014. The specimens were collected in sterile specimen jars. Only two stool specimens were collected and tested for both microscopy and culture. Food samples could not be collected and tested because there were no food leftovers.

### Environmental investigation

Water samples were collected for laboratory analysis. Checklists were used for the environmental and emergence preparedness and response assessments. The checklist for emergency preparedness and response was adopted from the Technical Guidelines for Integrated Disease Surveillance and Response (IDSR) in Zimbabwe [6].

Data collected from the study participants were entered into Epi Info™ to generate frequencies, means, proportions, risk ratios and attributable risk. Permission to carry out the study was obtained from the Bulawayo City Director of Health Services and Health Studies Office. Written informed consent was obtained from study participants. Confidentiality was assured and maintained throughout the study.

## Results

### Outbreak description

#### Study population

The response rate was 94 %. We interviewed a total of 74 BCC employees who attended the IRBM workhop at Ikhwezi Training Centre from 18 to 22 August 2014. Of these 34 (45.9 %) were males and 40 (54 %) were females. The median age in years was 45 (Q_1_ = 45; Q_3_ = 51). A total of 53 got ill after eating food served at the training centre on 20 August 2014. The majority were married (77 %) and had attained tertiary education (95.9 %) (Table 1).

**Table 1:** Demographic characteristics of study participants, Ikhwezi Training Centre, Bulawayo City, 2014

Variable	Category	Frequency (%) n = 74
Age		
	25–35	7 (9.4 %)
	36–45	31 (41.8 %)
	46–55	25 (33.7 %)
	56–65	11 (14.8 %)
Median age (years) 45 (Q_1_ = 40; Q_3_ = 51)		
Gender		
	Male	34 (45.9 %)
	Female	40 (54 %)
Marital status		
	Married	57 (77 %)
	Single	13 (17.6 %)
	Widowed	2 (2.7 %)
	Cohabiting	2 (2.7 %)
Level of education		
	Primary	0 (0)
	Secondary	3 (4.1 %)
	Tertiary	71 (95.9 %)

#### Place

The outbreak occurred at Ikhwezi Training Centre in Bulawayo City after the study participants had eaten the food that was served at 1300 h during lunch on 20 August 2014.

#### Epidemic curve

**Fig. 1:**
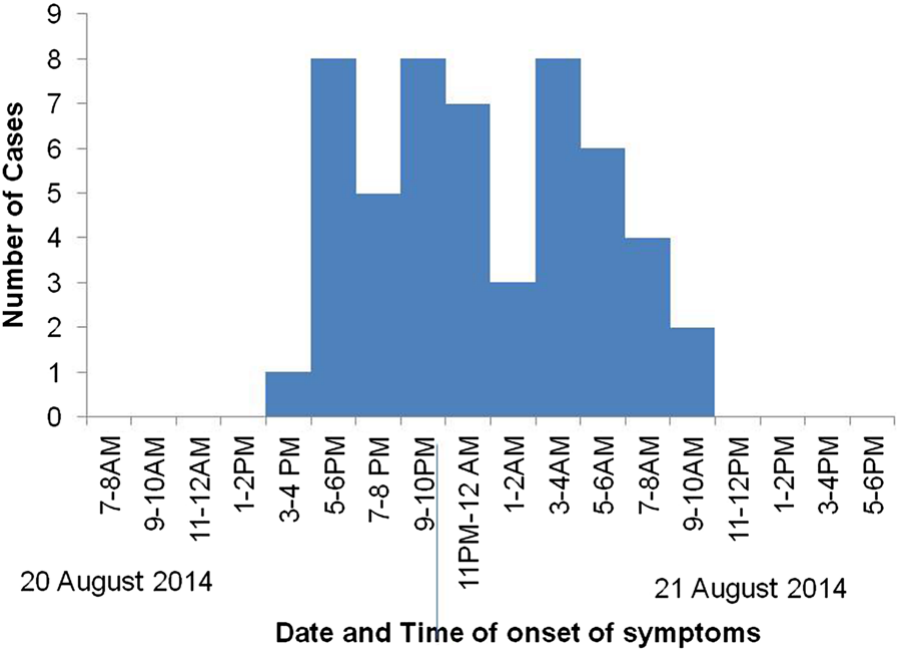
An epidemic curve of the food poisoning outbreak at Ikhwezi Training Centre 20–21 August 2014

Figure 1, illustrates the onset of illness over time. The onset of Illness for the first case was reported between 3 and 4 p.m. on the 20th of August 2014 after having eaten lunch served at 1300 h and cases continued to be reported between 5 and 6 p.m. reaching the first peak. There was a decline between 7 and 8 p.m. followed by a rise between 9 and 10 p.m. reaching the second peak. A decline was also noted between 1 and 2 a.m., followed by a rise between 3 and 4 and reaching the third peak. Cases began to decline until 10 a.m. on August 21, 2014 August. The median incubation period from the time lunch was served was 11 h (Q1 = 7; Q3 = 16).

#### Illness characteristics

**Fig. 2:**
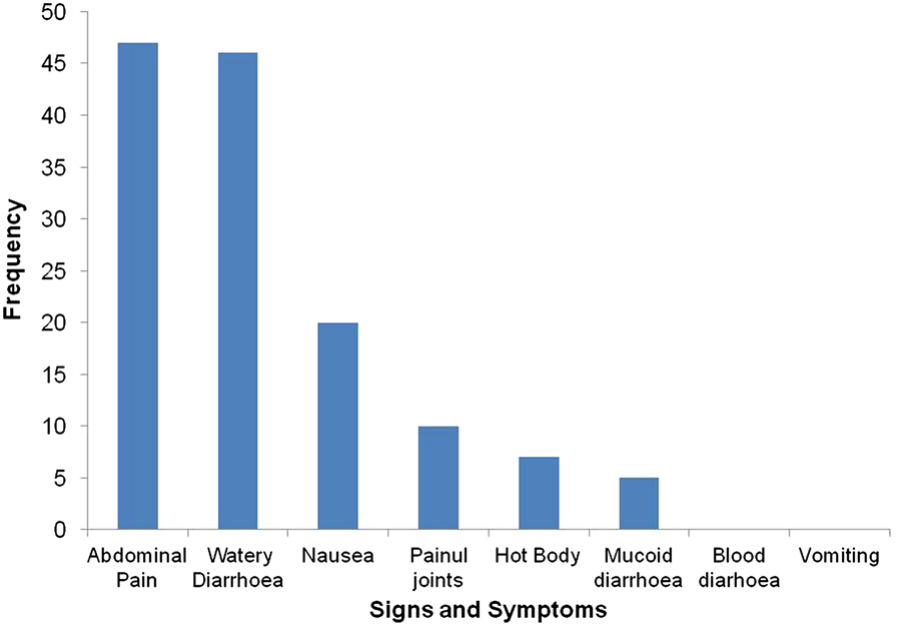
Food poisoning signs and symptoms at Ikhwezi Training Centre, Bulawayo City, 2014

Figure 2, is a summary of the signs and symptoms presented by the 53 employees who reported that they were ill after eating the food at the Ikhwezi Training Centre. The majority, 47 (88.7 %) presented with abdominal cramps, 46 (86.8 %) watery diarrhoea, 20 (37.7 %) with nausea, 10 (18.9 %) with painful joints, 7 (13.2 %) with hot body and 5 (9.4 %) presented with mucoid diarrhoea. Vomiting and bloody diarrhoea were not reported. The overall attack rate was 71.6 %.

### Analytic epidemiology

#### Significant risk factors for food poisoning

Bivariate analysis was used in analysing the factors associated with the outbreak. Multivariate analysis could not be performed with only one significant risk factor. The statistically significant risk factor for diarrhoea was the stewed chicken (RR = 2.52, 95 % CI 1.30–4.89) served during lunch at 1300 h at Ikhwezi Training Centre in Bulawayo on August 20, 2014. Drinking bottled water (RR = 0.67, 95 % CI 0.57–0.79) was statistically significant as a protective factor. Eating Sadza, rice, roasted chicken, green beans, green vegetables, carrots, cabbage, jelly, fruit cocktail, custard and ice cream were not associated with the food poisoning as indicated by the risk ratios which are not statistically significant. Drinking municipal water was protective but not statistically significant as shown in Table 2.

**Table 2:** Bivariate analysis of foods consumed during lunch on 20/08/2014 at Ikhwezi Training Centre, Bulawayo City, 2014

Food item	Consumed			Not consumed			RR	95 % CI
	Ill	Not ill	Attack rate (%)	Ill	Not ill	Attack rate (%)		
Sadza	35	13	72	18	8	69	1.05	0.77–1.43
Rice	34	13	72	19	8	70	1.02	0.76–1.39
Stewed chicken	47	9	84	6	12	33	2.52	1.30–4.89
Roasted chicken	23	13	64	30	8	79	0.81	0.60–1.09
Green beans	19	8	70	34	13	72	0.97	0.72–1.32
Green veg.	31	14	69	22	7	76	0.91	0.68–1.21
Carrots	14	6	70	39	15	72	0.97	0.70–1.35
Cabbage	21	7	75	32	14	70	1.08	0.81–1.44
Jelly	25	11	69	28	10	74	0.94	0.71–1.26
Fruit cocktail	10	4	71	43	17	72	0.99	0.69–1.44
Custard	28	10	74	25	11	69	1.06	0.80–1.42
Ice cream	21	7	75	32	14	70	1.08	0.81–1.44
Bottled water	43	21	67	10	0	100	0.67	0.57–0.79
Municipal water	5	3	63	48	18	72	0.86	0.49–1.50

The excess risk of illness among BCC employees who ate stewed chicken which was served at Ikhwezi Training Centre on 20 August 2014 during lunch was 50.59 per 100 individuals and was statistically significant as indicated by the confidence interval. The excess risk of illness could be eliminated if they had not eaten stewed chicken.

The response rate for stool sample collection was 25 %. We managed to collect two stool specimens out of the planned eight samples. Stool specimens collected and analysed were negative for both microscopy and culture. Staphylococcus aureus was isolated from the hands and nails of food handlers (Table 3). Water samples showed no bacterial growth.

**Table 3:** Laboratory swab results for the food poisoning outbreak at Ikhwezi Training Centre, Bulawayo City, 2014

Sample	Microorganism obtained
Hands of food handler 1	No growth
Nails of food handler 1	Staphylococcus aureus
Hands of food hander 2	Staphylococcus aureus
Nails of food handler 2	Staphylococcus aureus
Hands of food handler 3	No growth
Nails of food handler 3	Staphylococcus aureus
Hands of food handler 4	Staphylococcus aureus
Nails of food handler 4	Staphylococcus aureus
Hands of food handler 5	Staphylococcus aureus
Nails of food handler 5	Staphylococcus aureus
Fork	No growth
Plate	No growth
Knife	No growth
Food tray	Insignificant growth

### Epidemic preparedness and response

Bulawayo City was prepared for the outbreak. The rapid response team responded after 5 h following receipt of the outbreak report against the stipulated timeframe of within 48 h. The team line listed all those who had diarrhoea. Stool specimens were collected however there was a day’s delay in the receipt of specimen results. The delay was due to workload at the laboratory.

Bulawayo City was adequately stocked with oral rehydration sachets (8000) and ringers lactate (130 × 1000 mls). Normal saline (1240 × 500 mls), and Intravenous cannulas, 20G (130), 18G (130), and 16G (130) were available. Oral drugs such as Cotrimoxazole (2655 × 500 tins), Metronidazole (8450 tablets) were available. Essentials such as latex gloves (8000 pairs) and aprons (80) were available. Flyers (10,000) and posters (10,000) related to diarrhoea were available. Disinfectants available were High Test Hypochlorite (HTH) (70 kgs) and chloride of lime (50 kgs).

### Environmental health audit

#### Food handlers

Five food handlers were involved in food preparation for the council employees attending the workshop at Ikhwezi Training Centre. Only one was a trained cook. The rest revealed that they were not trained in food handling. All of them had clean uniforms. All of them reported that they did not have medical examination certificates. The catering company was not licensed with the Health Services Department.

#### Kitchen

The kitchen was dirty and the dining room was fairly clean. Hand washing facilities with solid hand washing soap were available. Two taps were functional and running water was available. Hot water was not provided in the kitchen.

#### Toilets

There were four (two male and two female) functional toilets with a total of 16 water closets. The toilets were provided with municipal water. Two urinals were available for males. Hand washing facilities and solid soap were provided in the toilets. The toilets were adequate for the numbers of people present although they were dirty.

#### Storage

Perishable foods were stored in a deep freezer and non perishable foods were stored in buckets and containers. The deep freezer was clean.

#### Waste management

Refuse and waste were disposed in a bin. The food handlers reported that the leftover food was given away to staff and relatives. Upon investigation, one staff member and a relative who were given the food leftovers did not became ill.

## Discussion

The outbreak affected a large number of people with an overall attack rate of 71.6 %. The epidemic curve suggests a point source outbreak. The short incubation period was suggestive of an enterotoxin producing bacterium such as staphylococcus aureus whose incubation period is 2–36 h [7].

Food leftovers could not be found for bacteriological analysis. This was because it had been given away to one staff member and a relative. None of them became ill. Food handlers were asked to always keep a plate of food leftovers for some time before disposal so that in case of an outbreak a food sample will be available for analysis. These findings are similar to a study by Bangure et al. [8] in 2012 on food poisoning among census enumerators in Gokwe South District, where they found out that staphylococcus was isolated from the hands of food handlers although no leftover food could be sent for microbial analysis. The findings are also similar to a study by Moyo et al. [9] in 2004 on bacteriological assessment of the cleaning and disinfection efficacy at Midlands State University where they also found out that 40 % of the tested food handlers’ hands were contaminated with staphylococcus aureus. Staphylococcus aureus has been linked to several outbreaks. If food handlers carry an enterotoxin producing staphylococcus aureus they may contaminate the food and cause staphylococcus aureus food poisoning. Hand washing with soap has been demonstrated to reduce the risk of diarrhoea diseases.

In this study we found that the major signs and symptoms presented were abdominal cramps (88.7 %) and watery diarrhoea (86.8 %). Vomiting and bloody diarrhoea were not reported. This finding is similar to a gastroenteritis outbreak investigation in an airforce base in Western Greece, by Jelastopulu et al. [10] in 2006, where they found out that among the symptoms, the most prominent were watery diarrhoea (96 %) and abdominal pain (73 %). Symptoms of staphylococcus aureus food poisoning from other studies are similar to the symptoms presented in this outbreak. Contamination of food with staphylococcus causes gastrointestinal illness of sudden illness because the organism was found to be capable of producing an enterotoxin. The food normally implicated in staphylococcus food poisoning is meat and its products [9, 11, 12]. Our findings therefore suggest staphylococcus aureus food poisoning.

The stewed chicken that was served during lunch on 20 August 2014 at Ikhwezi Training Centre was implicated in the diarrhoeal disease outbreak. Contamination might have been caused by the staphylococcus aureus which was isolated from the hands of the food handlers. Enterotoxins may have been released during food handling and preparation.

In this study the water samples that were collected and analysed at Bulawayo City Laboratory were negative for both faecal and nonfaecal coli forms. We also found that drinking bottled water during lunch on 20 August2014 at Ikhwezi Training Centre was protective and statistically significant. Drinking municipal water during lunch on 20 August 2014 at Ikhwezi Training Centre was also protective although it was not statistically significant. This also ruled out a waterborne disease outbreak.

Majority (80 %) of the food handlers were not trained in food handling. Neither did they have the qualifications. This finding is similar to a study by Chihava et al. [13] in 2012 on factors contributing to biological diversity and load in Bulawayo City restaurants, where they found out that the majority of food handlers in Bulawayo restaurants were not trained and lacked the necessary qualifications in food handling. Food handlers are a crucial link in the food chain from farm to fork and as such they should be trained so that they improve their practices in food handling and preparation.

All the food handlers had no medical examination certificates. This finding is similar to a study by Dagnew et al. [14] in 2002 on the survey of nasal carriage staphylococcus aureus and intestinal parasites among food handlers working at Gondar University, Northwest Ethiopia, where they found that food handlers had no medical check-up.

The City Health Department responded well to the outbreak, line listing was done. However, there was a day’s delay in receiving the stool specimen results.

### Study limitations

Only two stool specimens were collected. This was because the rapid response team arrived at Ikhwezi Training Centre after 5 h following the report of the outbreak and managed to collect two samples from the only two council employees who still had diarrhoea.

### Recommendations

We therefore recommend the Senior Health Promotions Officer to intensify health education by distributing Information Education and Communication (IEC) materials to restaurants, hotels and food outlets in Bulawayo City. The Assistant Director Environmental Health Unit to train food handlers in basic food hygiene and safety, ensure that food handlers are medically examined regularly, advise Council to contract licensed catering companies for catering services during Council workshops at Ikhwezi Training Centre and intensify food sampling and swabbing as this will monitor the safety of food available for Bulawayo Market. Outbreak surveillance and reporting systems are to be strengthened inorder to quickly detect and report conditions of public health importance. The rapid response team should timely collect specimens during outbreaks. The Ministry of health to capacitate local government laboratories to carry out virological tests.

### Public health actions done

The following activities were done in order to control the outbreak:

Health Education was given to the BCC employees who were attending the workshop at Ikhwezi Training Centre. Health Education was given to the food handlers who were involved in food preparation. Food handlers were assessed for medical fitness. Council was advised to hire licensed catering companies for catering services. Food handlers in Bulawayo City have been trained on basic food hygiene and safety.

## Conclusions

This was a food poisoning outbreak. Stewed chicken was associated with this suspected food poisoning. Information from the epidemic curve and the short incubation period as well as the presenting signs and symptoms fit the profile of an enterotoxin producing bacteria like staphylococcus aureus which was isolated from the hands of the food handlers. Food contamination might have occurred during food handling and preparation. All the food handlers had no medical examination certificates. The Rapid Response team responded timeously to the outbreak and the department was adequately stocked with drugs and resources.

## Abbreviations

BCC: Bulawayo City Council
CDC: centres for disease prevention and control
FBDs: foodborne diseases
HTH: high test hypochlorite
IDSR: integrated disease surveillance and response
IEC: information education and communication
IRBM: integrated result based management
WHO: World Health Organisation
